# Domain-specific versus generalized cognitive screening in acute stroke

**DOI:** 10.1007/s00415-015-7964-4

**Published:** 2015-11-20

**Authors:** Nele Demeyere, M. J. Riddoch, E. D. Slavkova, K. Jones, I. Reckless, P. Mathieson, G. W. Humphreys

**Affiliations:** 1 Cognitive Neuropsychology Centre, Department of Experimental Psychology, University of Oxford, Oxford, OX1 3UD UK; 2 Acute Stroke Unit, John Radcliffe Hospital, Oxford University Hospitals NHS Trust, Oxford, UK

**Keywords:** Cognition, Stroke, Cognitive assessment, Neuropsychology

## Abstract

Cognitive assessments after stroke are typically short form tests developed for dementia that generates pass/fail classifications (e.g. the MoCA). The Oxford Cognitive Screen (OCS) provides a domain-specific cognitive profile designed for stroke survivors. This study compared the use of the MoCA and the OCS in acute stroke with respect to symptom specificity and aspects of clinical utility. A cross-sectional study with a consecutive sample of 200 stroke patients within 3 weeks of stroke completing MoCA and OCS. Demographic data, lesion side and Barthel scores were recorded. Inclusivity was assessed in terms of completion rates and reasons for non-completion were evaluated. The incidence of cognitive impairments on both the MoCA and OCS sub-domains was calculated and differences in stroke specificity, cognitive profiles and independence of the measures were addressed. The incidence of acute cognitive impairment was high: 76 % of patients were impaired on MoCA, and 86 % demonstrated at least one impairment on the cognitive domains assessed in the OCS. OCS was more sensitive than MoCA overall (87 vs 78 % sensitivity) and OCS alone provided domain-specific information on prevalent post-stroke cognitive impairments (neglect, apraxia and reading/writing ability). Unlike the MOCA, the OCS was not dominated by left hemisphere impairments but gave differentiated profiles across the contrasting domains. The OCS detects important cognitive deficits after stroke not assessed in the MoCA, it is inclusive for patients with aphasia and neglect and it is less confounded by co-occurring difficulties in these domains.

## Introduction

Following stroke, cognitive deficits are frequent [1–4], predictive of recovery [5–12] and interfere with rehabilitation (e.g. due to poor comprehension or spatial attention). Cognitive deficits after stroke are also associated with a reduced quality of life [13–15] and depression [8]. Due to their prevalence and importance, early detection is required to facilitate rehabilitation.

To facilitate early detection, short generalized cognitive screening tools are increasingly adopted. The Montreal Cognitive Assessment (MoCA) [16, 17] is one tool which is freely available and easy to administer, returning a pass/fail generalized cognition score. Though developed for dementia, the MoCA has been shown to have better sensitivity in detecting post-stroke cognitive impairments than the traditionally used Mini-Mental Status Examination (MMSE) [18–21]. However, neither the MMSE nor the MOCA assesses common post-stroke domain-specific impairments including aphasia, visual loss, visuo-spatial inattention (neglect), apraxia and reading/writing problems. Furthermore, performance on the tests that are included can be confounded by co-occurring problems. For example, arguably all of the MoCA subtests require substantial verbal abilities and aphasic patients will fail tests of non-language domains (e.g. memory) because of language impairments. Similarly, patients can fail subtests because they neglect one side of the page (e.g. in the trail making test).

Clinical guidelines emphasize the need to assess performance across different domains of cognition after stroke (e.g. “*attention, memory*, *spatial awareness, apraxia, perception*”*—UK National Institute for Clinical Excellence guideline for stroke care*, *2013*), highlighting the need for domain-specific cognitive assessments. Detailed neuropsychological examinations can detect specific cognitive impairments [2, 22]. Not surprisingly, when comparing a short MoCA screen to a detailed battery of neuropsychological assessments, the detection rate of cognitive problems was demonstrably lower in the MOCA [23]. However, detailed batteries are often impractical (not designed for acute stroke and very time consuming) and need trained examiners for administration, who cannot routinely see all patients.

A recent review and meta-analysis of test accuracy of cognitive screening tests concluded that there was no clearly superior screening test (comparing MoCA, ACE-R, MMSE and CAMCOG). It should be noted, however, that none of the screens were stroke-specific and the studies that were included focussed on generalized impairments, equating cognitive impairments to dementia. In addition, only 11 of the 35 included studies were conducted in acute stroke [24].

The Oxford Cognitive Screen—OCS [25] was specifically developed to measure domain-specific cognitive deficits in acute stroke. It provides a short cognitive screen covering five cognitive domains, including the assessment of important and commonly found stroke-specific cognitive problems, such as unilateral neglect, aphasia and apraxia. The reporting structure emphasizes the domain specificity of problems going beyond an overall pass/fail outcome. It also goes beyond other measures by being designed to avoid confounding effects within the separate cognitive domains providing ‘aphasia and neglect friendly’ measures of performance.

In this study, we compared domain-specific cognitive screening (OCS) with generalized screening provided through the MoCA, in an acute stroke population. We examined (1) how well the tools detected stroke-specific cognitive impairments, and (2) their clinical utility in terms of patient inclusion and generating accurate cognitive profiles for patients with co-occurring deficits.

## Methods

### Materials

The Oxford Cognitive Screen (OCS) is a recently developed stroke-specific cognitive screen (see [20] for normative data, validation and reliability and sensitivity measures of the OCS). The OCS is structured around five domains: (1) attention and executive function, (2) language, (3) memory, (4) number processing and (5) praxis. The tests were designed to be inclusive and uncontaminated by aphasia and neglect, when (respectively) language and spatial attention are not assessed. The test is freely available for clinical use and licensed through the University of Oxford’s technology transfer office (http://www.ocs-test.org). The OCS, as a domain-specific assessment, provides a ‘visual snapshot’ of a patient’s cognitive profile, for easy domain level (see [25]).

The MoCA is also freely available for clinical use and consists of a single A4 page. Though the MoCA contains some subsections, they are typically not separately marked (there are no sub-domain cut-offs). As a domain-general cognitive screen, the MoCA ultimately returns a pass/fail judgement, based on a single overall score. Permission was received from the MoCA team for its use in this research.

Both the OCS and MoCA are paper-and-pen-based assessments administered within 25 min., making them time-efficient and suitable for acute post-stroke assessment. In addition, both screening tools can be delivered at the bedside, are easy to administer and score, and can be filed into the patients’ clinical notes.

### Participants

A consecutive sample of 200 stroke patients was recruited from the acute stroke unit at the John Radcliffe Hospital, Oxford and the University Hospital Coventry and Warwickshire. Inclusion criteria were: patients (1) were within 3 weeks of a confirmed ischaemic or haemorrhagic stroke diagnosis by clinicians, (2) were able to concentrate for 15 min (as judged by the multidisciplinary care team) and (3) were able to give informed consent (which could be witnessed in case of language difficulties or motor difficulties with signing the consent forms).

The mean age of the patient sample was 70.5 (SD = 14.7) years, and the average time of assessment was 6.1 days post-stroke (SD = 4.4). Further sample characteristics with regard to gender, handedness and lesion aetiology and lateralisations are given in Table 1. Lesion lateralisations for the sample were: 78 unilateral left hemisphere, 98 unilateral right hemisphere, 18 bilateral and 6 unclear from scan (classifications taken from the medical notes and confirmed by CT scan).

**Table 1:** Patient sample characteristics for the consecutive sample of 200 acute stroke patients, for whom cognition was assessed after an average of 6.1 days (SD = 4.4)

Sample characteristic	Category	Proportion of patients (*N* = 200)
Gender	Male	0.55
	Female	0.45
Handedness	Left	0.07
	Right	0.92
	Ambidextrous	0.01
Aetiology	Haemorrhage	0.10
	Ischaemia	0.90
Vascular territory affected	Lacunar infarcts	0.24
	ACA	0.15
	LSA	0.13
	MCA	0.26
	PCA	0.15
	PICA	0.05
	Unclear	0.03
Lesion lateralisation	Unilateral left hemisphere	0.39
	Unilateral right hemisphere	0.49
	Bilateral	0.09
	Unclear from scan	0.03

### Procedure

Once informed consent was given, participants completed the two cognitive screens with a trained researcher, using a randomised ordering of the tests. There was a maximum of 5 days between assessments, with 90 % of patients assessed on both screens within 24 h (average 1 days, SD = 1.3).

Two patients were excluded from the analysis as they had a further serious medical event before the second cognitive assessment could be completed.

### Standard protocol approvals, registrations, and patient consents

This study was approved by the National Research Ethics Service (Ref: 11/WM/0299; Protocol RP-DG-0610-10,046). Written or witnessed informed consent was obtained from all participants.

## Results

### Inclusivity

Table 2 reports the inclusion rates and reasons for non-completion separately for the OCS and MoCA sub sections. The reasons for non-completion varied from poor vision to difficulties with understanding instructions and practical problems such as interruptions and running out of time. In the MoCA, all but the initial section (“visuospatial/executive”—5 points of the total 30), requires expressive speech. From Table 2, we note that in our sample, 14 patients (7 %) were severely aphasic (either expressive or global aphasia) resulting in a complete loss of speech and therefore an inability to complete all but the three initial MoCA subtests (amounting to a maximum score of 5/30). Note that all the included patients were able to give informed consent, and therefore the acute sample presented here excludes patients with severe global aphasia.

**Table 2:** Inclusion and reasons for not testing on all subtests of the OCS and MoCA

	Measure	Inclusion rate (%)	Completed: not completed due to problems with:								
			*N*	Speech	Comprehension	Vision	Motor	Time	Fatigue	Illiterate	Examiner error
OCS vs MoCA											
Language	Picture naming	99	196		1	1					
	Semantics	99	196		1	1					
	Sentence reading	93	184	9	1	3				1	
Memory	Orientation	99	197		1						
	Recall and recog	99	197		1						
Number	Number writing	97	193		2		2			1	
	Calculation	99	196		2						
Perception	Visual field	98	195		3						
Spatial attention	Hearts cancellation	91	181		9	6	1		1		
Praxis	Imitation	98	195		2	1					
Controlled attention	Executive task	95	188		5	3	1	1			
MOCA											
Visuo-Spatial	Trails	94	186		8	3	1 (optic ataxia)				
	Cube	94	187		6	3	2				
	Clock	95	188		6	2	1 (optic ataxia)			1	
Naming	Picture naming	94	186	11		1 (blind)					
Memory	Word encoding	93	185	11	1						1
Attention	Digits	93	184	11	3						
	Tap to A	93	185		13						
	Serial 7s	93	184	11	3						
Language	Repetition	93	184	11	3						
	Fluency	93	184	11	3						
Abstraction	Abstraction	93	184	11	3						
Delayed recall	Delayed recall	93	184	11	3						
Orientation	Orientation	93	184	11	3						

Importantly, not only could the aphasic patients be included when tested using the OCS, they returned scores in the different domain subsections outside of language production. For the memory domain, all of these patients returned scores for orientation and verbal recognition memory, with 50 % (*N* = 7) demonstrating perfect scores on the orientation questions with forced-choice testing, and only two patients scoring 1 or 0/4. Similarly, for the OCS domain of praxis, all the patients excluded on the majority of MoCA were able to complete the task and returned scores, with 36 % (*N* = 5) not demonstrating any praxic impairment.^1^ In the number domain, again all the patients excluded on the MoCA produced scores, and although all bar 1 patient failed the number writing task, 6 (43 %) passed the multiple choice calculation test. In the attention domain, 71 % (10) of the excluded patients generated a spatial attention score on the OCS (four failed to do so due to complex instruction comprehension problems). Of these ten, four demonstrated no impairment, four presented with right egocentric neglect, one with right allocentric neglect and one with both ego- and allocentric neglect. Thus, the OCS can be used to detect neglect in aphasic patients. For the test of executive function, 8 (57 %) of the excluded patients returned scores, and 50 % (4) were not impaired on the executive score.

### Domain specificity

MoCA is a domain-general cognitive screen, summing up the different sections of the task into a single score. In contrast, the OCS is divided into separate cognitive domains, each with associated normative data. Here, we examined the differences between MoCA and OCS with respect to domain specificity in common post-stroke impairments.

The OCS provides domain-specific information on common post-stroke cognitive impairments including neglect, apraxia, number and reading/writing ability—none of which are evaluated in the MoCA. Language comprehension is assessed in the semantics task of the OCS, reading in the sentence-reading task. Writing to dictation is assessed in the number writing task. Neglect is assessed in detail in the Broken Hearts task, with measures given for both egocentric and allocentric neglect. Apraxia is assessed through the imitation of meaningless gestures. The high incidences of these specific impairments are demonstrated in Table 3.

**Table 3:** Incidence of impairments in a consecutive sample of acute stroke patients, for the overall sample, and for left hemisphere damage (LHD) and right hemisphere damage (RHD) separately

Screen	Domain	Measure	Overall (%)	LHD (%)	RHD (%)	Fisher’s exact
OCS	Language	Picture naming	29.7	36.0	26.0	0.18
		**Semantics**	**7.1**	9.1	7.3	0.78
		**Sentence reading**	26.0	38.4	17.7	<0.01**
	Memory	Orientation	16.2	18.2	15.4	0.68
		Recall and recog	26.4	40.8	13.4	<0.01**
	**Number**	**Number writing**	**31.1**	42.5	22.9	<0.01**
		Calculation	14.2	22.4	6.2	<0.01**
	**Perception**	**Visual field**	**15.9**	13.3	19.6	0.31
	**Spatial attention**	**Spatial neglect**	**39.8**	30.0	47.8	0.024*
		**Object neglect**	**23.2**	18.6	31.1	0.15
	**Praxis**	**Imitation**	**27.6**	29.0	25.8	0.73
	Controlled attention	Executive task	48.9	47.2	51.6	0.86
MoCA	Overall score	Cut-off = 26	76.26	77.92	73.20	0.49
		<20	38.89	44.16	30.93	0.08
		<15	25.17	41.67	12.67	<0.001**

In bold: areas in OCS not unambiguously assessed in MoCA

Fisher’s exact tests comparing frequencies of impaired vs not impaired in LHD and RHD groups

* Significant at 0.05 two-tailed criterion

** Significant 0.01 two-tailed criterion

In addition to overall incidences, we calculated the levels of impairment on the different subtasks for patients with unilateral left or unilateral right hemisphere lesions (Table 3). Low scores on MoCA were more common in left hemisphere patients (Fisher’s exact *p* < 0.001). In contrast, the OCS presented a profile more differentiated according to the nature of the cognitive domain being tested. While the language, number and verbal memory were more commonly impaired after left hemisphere damage, this was not the case for the praxis, spatial, and executive attention domains.

### Cognitive profiles

Using the proposed single value cut-off of 26 for the MoCA, 76.3 % of our sample of patients were impaired. Of the 47 patients scoring above 26 on MoCA, 80.9 % (*N* = 38) demonstrated at least one domain impairment on the OCS. Overall, just 14.1 % of the total sample of patients demonstrated no impairments on any of the five cognitive domains assessed in the OCS. Of these 28 patients, 64.3 % scored below the MoCA cut-off (*N* = 18). This gives the OCS (in comparison with the MoCA) a sensitivity of 87.7 %, in contrast, when comparing the MoCA with the OCS, the sensitivity of the MoCA is 78.2 %.^2^

However, pass/fail rates per se carry little information about the nature of the impairment in a given patient. Instead, for a comparison of a domain general with a domain-specific screen, it is of more interest to determine which cognitive domains are failed in the OCS, despite being ‘passed’ in the MoCA, and vice versa. Of the 47 patients who passed the MoCA, 27.7 % (*N* = 13) demonstrated an impairment on just one task in the OCS and 51.1 % (*N* = 24) failed more than one subtask (10 were impaired in two subtasks, 10 in 3, and 4 in 4 or more). Table 4 demonstrates which OCS subtests were failed despite the patient passing on the MoCA. Of note is that these patients had deficits in abilities not evaluated on the MoCA, with 50 % showing spatial neglect, as well as large proportions demonstrating difficulties with reading, writing and executive tasks (see Table 4).

**Table 4:** OCS task impairment incidences of patients with MoCA > 25 (*N* = 36)

Domain	Task	*N*	% impaired
Language	Picture naming	1	2.78
	Semantics	0	0.00
	**Sentence reading**	6	**16.67**
Memory	Orientation	1	2.78
	Recall and recog	1	2.78
Number	**Number writing**	6	**16.67**
	Calculation	1	2.78
Perception	Visual field	5	13.89
**Spatial attention**	**Spatial neglect**	18	**50.00**
	**Object neglect**	10	**27.78**
**Praxis**	**Imitation**	6	**16.67**
**Controlled attention**	**Executive task**	**12**	**33.33**

Tasks and domains in bold denote areas of cognitive impairments hat are not assessed in MoCA

Of the 18 patients who failed the MoCA, but had no impairments on OCS, 66.7 % (*N* = 12) scored in a range between 23 and 25 on the MoCA and thus were close to the ‘pass’ level and would be considered to have a mild deficit.

The OCS provides a cognitive profile. Within this profile, the co-occurrence of impairments is common [26], though domains also dissociate. In our sample, 85 % of the patients were impaired on at least one cognitive domain in the OCS; 25 % were affected in only one sub-domain, 24 % in two, 14 % in three, 14 % in four, and 8 % in five sub-domains.

To further investigate associations of performance across all subtasks in a consistent manner, all outcomes were transformed to a categorical outcome (pass or fail depending on the task-specific cut-off values). Table 5 presents Cramer’s V (phi) values denoting the strength of association between each pairing of subtasks based on the categorical data. High strengths of association were noted between the language, number, praxis and memory domains. In addition, an association was found between the controlled and spatial attention tasks, which did not associate strongly with the other three domains.

**Table 5:** Strength of association (Cramer’s V) between categorical outcomes (pass/fail) on each of the OCS subtasks

		Language			Memory		Number		Spatial attention		Praxis
		Picture naming	Semantics	Sentence reading	Orientation	Recall and recog	Number writing	Calculation	Spatial neglect	Object neglect	Imitation
Language	Picture naming	–									
	Semantics	0.280**	–								
	Sentence reading	0.288**	0.194*	–							
Memory	Orientation	0.296**	0.097	0.181*	–						
	Recall and recog	0.440**	0.220*	0.206*	0.152 *	–					
Number	Number writing	0.309**	0.251**	0.321**	0.313**	0.241**	–				
	Calculation	0.324**	0.125	0.447**	0.143*	0.356**	0.310**	–			
Spatial attention	Spatial neglect	0.079	0.044	0.013	0.074	0.032	0.09	0.058	–		
	Object neglect	0.067	0.055	0.096	0.083	0.143	0.109	0.045	0.168*	–	
Praxis	Imitation	0.325**	0.277**	0.204*	0.233**	0.301**	0.314**	0.235**	0.185*	0.077	–
Controlled attention	Executive task	0.085	0.052	0.01	0.13	0.142	0.157*	0.104	0.153*	0.109	0.169*

Values in bold denote highly significant assocations

* Denotes *p* < 0.05

** Denotes *p* < 0.001

### Confounds

Aside from the inclusion of severely aphasia patients, milder language impairments commonly found after left hemisphere stroke (e.g. anomia, language apraxia, and reading and writing impairments) may impact on a participant’s performance on a generalized cognitive screen such as the MoCA. Indeed, as we have noted, patients with left hemisphere damage scored lower on the MoCA (even with globally aphasic patients excluded; see Table 3) which may reflect these other language impairments.

To consider a group of moderate language impairment patients, we took patients who failed^3^ both language tasks on the MoCA (sentence repetition and fluency, *N* = 60) and at least one of the two language tasks in OCS (picture naming and sentence reading) (*N* = 43/60). The OCS criterion was added because failing the two MoCA language tasks may arguably be due to non-linguistic impairments (e.g. sentence repetition requires working memory [27]; fluency tasks demand working memory too along with cognitive inhibition to refrain from repeating words [28, 29].^4^

The performance of this group of moderate language impaired patients (*N* = 43) in the non-language domains is given in Table 6. With the exception of the visual fields test, all patients with a moderate language impairment (operationally defined), performed worse than those patients who had perfect scores on all language tasks in the OCS and MoCA (all Fisher’s exact comparisons *p* < 0.01). This may either reflect a generalized cognitive impairment profile in this group, or a language contribution aspect in the understanding of the non-language tasks in both the OCS and MoCA.

**Table 6:** Patients with language impairments: performance on non-language domains

	Moderate lang impairment group		No language impairment group	
	*N* (pass)	%	*N* (pass)	%
Total	43		47	
OCS subtasks				
OCS comprehension	34	79.1	47	100.00
OCS orientation	25	58.1	46	97.87
OCS vis field	32	74.4	40	85.11
OCS number write	15	34.9	42	89.36
OCS calculation	28	65.1	47	100.00
OCS hearts	13	30.2	28	59.57
OCS praxis	21	48.8	38	80.85
OCS VerbalMemory	18	41.9	44	93.62
OCS TaskSwitching	21	48.8	39	82.98
MoCA				
Overall score (>26 cut-off)	0	0.0	20	42.55
Overall score (>20 cut-off)	5	11.6	43	91.49
MoCA orientation (min 5/6)	15	34.9	46	97.87
MoCA trails	12	27.9	33	70.21
MoCA word memory (min 4/5)	1	2.3	20	42.55
MoCA serial 7 subtraction	5	11.6	37	78.72

Moderate language impairment group assigned if failing the MoCA language subsection (sentence repetition and fluency) as well as at least one of the OCS language tasks (sentence reading and picture naming)

No language impairment group assigned if passing all language tasks in MoCA and OCS

However, the purpose of this subgroup analysis was to compare the performance on similar measures within OCS and MoCA for patients with a language impairment, to establish whether the OCS is less confounded by language demands (as it was designed to be). For example, the orientation task is arguably similar in content in both the OCS and MoCA; however, the OCS allows multiple choice pointing responses to reduce the language demands. In the mild aphasic patients, this led to higher pass rates for the OCS orientation test compared to the equivalent subtest in the MoCA (42 vs 65 % impaired, one-tailed Fisher’s exact probability, *p* 0.026). Comparisons of the OCS trail making test (which uses non-verbal shapes) with the MoCA equivalent (which uses letters and numbers) again reveal a significantly better performance in the OCS (51 vs 78 % impaired, one-tailed Fisher’s exact *p* = 0.038). To demonstrate that these differences reflect the added language requirements rather than the overall difficulty of the tests, we reviewed the patients in the sample who scored perfectly on all the language domain subtests (MoCA and OCS —*N* = 47). Here, no differences in performance on the two comparable orientation tasks were found (2 % impaired in both OCS and MoCA), nor were any difference in impairment rates on the OCS vs MoCA trail making subtests noted (Fisher’s exact *p* = 0.22). Other tasks, such as the verbal memory and calculation tasks also have equivalents in MoCA, but these have significantly higher pass rates for both the subgroup of patients with and without language impairments (multiple choice calculation in OCS vs serial subtraction of 7 s in MoCA, Fisher’s exact *p* < 0.01 in both groups and verbal memory free recall in MoCA vs MCQ recognition in OCS, Fisher’s exact *p* < 0.01 in both groups). This simply demonstrates that these MoCA subtasks are arguably more demanding outside of language demands.

In sum, the performance on equivalent trail making and orientation tasks indicates that mild language impairments are more likely to impact on these similar tests in the MoCA than the OCS, confirming the successful attempt by the OCS to maximise the inclusion of patients with language impairments through reducing language demands on the cognitive domain subtests not assessing language. The results also further highlight the confounding effects of language impairments on the MoCA tasks and its return of a single overall score.

We conclude that failures on the putative non-language tests in the MoCA can reflect impaired language rather than a true deficit in these other domains.

In addition to the confounding effects of language, test performance can also be modulated by the presence of unilateral neglect. Consider the trails test. The MoCA letter/number alternating trails task is positioned in the top left corner of the page—a location that may be prone to left neglect (neglect being more likely in right than left hemisphere patients; e.g. [11]). In contrast, the OCS trail making task has baseline and switching tests using centrally positioned shapes (triangles and circles). In OCS, performance in the baseline is subtracted from that in the switch condition to reduce contamination from neglect and motor slowing. 180 patients completed both of these trails tasks. To have a range of scores for comparison, the MoCA test was re-scored by giving a point per correct connection (range 0–7, rather than the simple pass–fail as used clinically). 51 patients failed the MoCA trails and passed the OCS trails. Of those, 73 % failed to make a mark towards the most left elements on the MoCA trail, and 61 % demonstrated neglect on the OCS Broken Hearts test, suggesting at least partial contamination on the MoCA trails by visual neglect.

## Discussion

We compared the use of the OCS and the MoCA as neuropsychological screening tools for acute stroke patients. The data showed that, overall, the OCS had higher sensitivity than the MoCA in detecting cognitive impairments (88 vs 78 %). The OCS also detected significant numbers of patients with deficits in neglect, apraxia, reading, writing and number processing that went undetected using the MoCA. Previous work has shown that these cognitive impairments (e.g. in neglect or apraxia) are important predictors of outcome after stroke, highlighting the importance of being able to detect the presence of these deficits early [12, 26, 30]. These stroke-specific deficits can be screened efficiently and briefly using the OCS, even in acute stroke settings.

In addition to this, many patients who were excluded from the vast majority of tests on the MoCA due to poor spoken output could return scores on the sub-domains of the OCS, and in some instances the patients did not present with deficits under the aphasia-friendly test conditions (e.g. when measuring memory or executive functions). This means that the OCS can return a higher inclusion rate on the testing of acute stroke patients and the OCS can also highlight areas of strength in a patient, which would not otherwise be measured (e.g. when a patient has spared memory despite a language problem). As well as being less confounded by severe aphasia, our analysis also indicates that putatively non-language tests on the MoCA (e.g. the orientation and executive function trails tests) were more likely to be disrupted by milder language impairments than the equivalent assessments on the OCS. Again, the design properties of the OCS (forced-choice testing, using non-linguistic material) helped to reduce the confounding effects of language impairment.

In addition to the confounding effects of language, test performance after stroke can also be modulated by unilateral neglect (which was present in 40 % of our sample). Our analysis of the trails test in the MOCA indicated that a substantial proportion of patients failed this due to left neglect while being able to pass the equivalent test in the OCS. We attribute this to the OCS using a central array of stimuli (rather than placing stimuli on the left side of space) and emphasizing the contrast between switch and baseline conditions, which can subtract out effects of neglect.

One final crucial contrast between the two cognitive screening tools is that the MoCA is typically used to provide a pass or fail classification. In contrast to this, the OCS has a domain-specific reporting system with a visual reporting procedure that facilitates easy interpretation of impairments at the domain level. Given that the domain-level deficits in stroke patients are targeted by distinct therapies, (e.g. speech therapy for language impairments, occupational therapy for problems such as apraxia and neglect), domain-level reporting is likely to be important for rapid referral into the appropriate rehabilitation stream. In addition, the domain-specific assessment meets the guidelines for stroke screening as proposed by the UK National Institute for Clinical Excellence (NICE) [31].

### Practical issues

Our study has demonstrated that the OCS performs well against the MOCA, providing a better coverage of cognitive problems frequently encountered in stroke survivors, having increased sensitivity and reduced contamination by aphasia and neglect. To assess the practical application of the OCS, we surveyed 38 clinical professionals regularly using the OCS in NHS settings. We found that all experienced users completed the test within 25 min. Practitioners typically trained by reading the manual and practicing the test on a colleague and 55 % also watched the 15 min online demonstration video (http://www.ocs-test.org). Unlike the MOCA, the OCS does use several pages for the stimuli and test instructions, which help to clarify the tests for administrators and patients and which enable the OCS to de-confound effects of neglect. All surveyed users reported that the OCS was practical to use.

## Conclusion

In conclusion, the results indicate the OCS is a practical and sensitive tool for detecting and reporting important domain-specific cognitive problems after stroke. It maximises inclusion by being designed to reduce effects of aphasia and neglect. In these aspects, the OCS goes beyond measures derived from short dementia screens.

## Footnotes

1 Note that there is a long known link between aphasia and apraxia [26]; our findings concur with previous findings that most patients who demonstrate praxis problems will also have a dysphasia, though not necessarily the other way around.

2 We note that a ‘standard of truth’ does not exist for assessments of cognition. Here, we simply assess the sensitivity of the OCS relative to a current gold standard of clinical practice, the MOCA.

3 Although MoCA sentence repetition does not have task-specific cut offs, we defined failing the task if the participant made errors on at least one of the two sentence repetitions.

4 Fluency tasks are often used as part of assessment of organisational strategy within executive functioning (e.g. in DKEFS [29] see also the Addenbrooke’s Cognitive Examination [30].
